# HIV-1 Nef targets calnexin: a novel mechanism behind Nef effects on host cell and viral proteins

**DOI:** 10.1186/1742-4690-10-S1-P8

**Published:** 2013-09-19

**Authors:** Lucas Jennelle, Ruth Hunegnaw, Dmitri Sviridov, Michael Bukrinsky

**Affiliations:** 1 George Washington University School of Medicine and Health Sciences, Washington, DC, USA; 2 Baker IDI Heart and Diabetes Institute, Melbourne, Victoria, Australia; 3 University of Virginia, Charlottesville, VA, USA

## 

HIV-1 Nef is a pleiotropic modulator of various cellular signaling proteins and cell surface receptors. It conditions cells for viral replication by aiding in immune evasion and priming T-cell activation, and also enhances HIV-1 infectivity by a poorly defined mechanism. Most of described effects of Nef, such as downregulation of CD4 and MHC I, involve Nef binding to the target protein and directing it to a degradation pathway. We have demonstrated previously that Nef causes degradation and functional impairment of ABCA1, the major cellular cholesterol transporter, an effect that likely contributes to low HDL levels and high risk of atherosclerosis in HIV-infected patients. Surprisingly, direct interaction between Nef and ABCA1 was not required for ABCA1 inactivation. Here we investigated the mechanism of this Nef activity using proteomics, co-immunoprecipitation, mutagenesis, confocal microscopy, and subcellular fractionation approaches. These studies identified calnexin, an endoplasmic reticulum (ER) resident lectin-like chaperone, as an essential partner of ABCA1 and a target of Nef, and characterized Nef- calnexin interaction and its effects on interactions between calnexin and its other partners, such as gp160. We demonstrated that Nef binds to calnexin and increases affinity and enhances interaction of calnexin with HIV-1 gp160, but disrupts calnexin interaction with ABCA1. Interaction with calnexin is essential for functionality of both these proteins, as knock-down of calnexin led to reduced infectivity of HIV-1, as well as to defective cholesterol efflux. However, gp160 and ABCA1 interacted with calnexin differently: while gp160 binding to calnexin was dependent on glycosylation, glycosylation was of little importance for interaction between ABCA1 and calnexin. Thus, Nef binding appears to induce a conformational change in calnexin that stimulates glycosylation-dependent interaction of calnexin with gp160 at the expense of glycosylation-independent interaction with ABCA1. This study identifies a novel mechanism for Nef-dependent inactivation of ABCA1, provides an additional explanation for the effect of Nef on HIV-1 infectivity, and suggests a number of other potential targets of this new Nef activity.

